# Comparison of Femoral and Axillary Artery Cannulation in Acute Type A Aortic Dissection Surgery

**DOI:** 10.21470/1678-9741-2018-0354

**Published:** 2020

**Authors:** Orhan Gokalp, Levent Yilik, Hasan Iner, Nihan Karakas Yesilkaya, Yuksel Besir, Sahin Iscan, Bortecin Eygi, Ali Gurbuz

**Affiliations:** 1 Department of Cardiovascular Surgery, Izmir Katip Celebi University, Faculty of Medicine, Izmir, Turkey.; 2 Department of Cardiovascular Surgery, Izmir Katip Celebi University, Ataturk Education and Research Hospital, Izmir, Turkey.

**Keywords:** Aneurysm, Dissecting, Catheterization, Vascular Surgical Procedures, Surgeons

## Abstract

**Introduction:**

One of the most important points of the acute type A aortic dissection surgery is how to perform cannulation regarding cerebral protection concerns and the conditions of arterial structures as a pathophysiological consequence of the disease.

**Objective:**

In this study, femoral and axillary cannulation methods were compared in acute type A aortic dissection operations.

**Methods:**

The study retrospectively evaluated 52 patients who underwent emergency surgery for acute type A aortic dissection. Patients without malperfusion according to Penn Aa classification were chosen for preoperative standardization of the study groups. The femoral arterial cannulation group was group 1 (n=22) and the axillary arterial cannulation group was group 2 (n=30). The groups were compared in terms of perioperative and postoperative results.

**Results:**

There was no statistically significant difference in terms of preoperative data. In terms of postoperative parameters, especially early mortality and new-onset cerebrovascular event, there was no statistically significant difference. Mortality rates in group 1 and group 2 were 13.6% (n=3) and 10% (n=3), respectively (*P*=0.685). Postoperative new-onset cerebral events ratio was found in 5 (22.7%) in the femoral cannulation group and 6 (20%) in the axillary cannulation group (*P*=0.812).

**Conclusion:**

Both femoral and axillary arterial cannulation methods can be safely performed in patients with acute type A aortic dissection, provided that cerebral protection strategies should be considered in the first place. The method to be performed may vary depending on the patient’s current medical condition or the surgeon's preference.

**Table:** 

Abbreviations, acronyms & symbols	
ACP	= Antegrade cerebral perfusion
ASA	= American Society of Anesthesiologists
ATAAD	= Acute type A aortic dissection
CBP	= Cardiopulmonary bypass
CC	= Cross-clamp
CT	= Computed tomography
EJCTS	= European Journal of Cardiothoracic Surgery
EuroSCORE	= European System for Cardiac Operative Risk Evaluation
MACCEs	= Major adverse cardiac and cerebrovascular events
ICU	= Intensive care unit
ICVTS	= Interactive Cardiovascular and Thoracic Surgery
TCA	= Total circulatory arrest

## INTRODUCTION

Acute type A aortic dissection (ATAAD) is a catastrophic condition that is extremely deadly unless it is intervened. According to current guidelines, the gold standard in the treatment of this condition is the surgical intervention^[1]^. Despite being a surgical procedure that has been performed for many years, the 30-day mortality rate of this procedure is still 20%^[2]^. The most important reason for mortality and morbidity in both preoperative and postoperative periods is the organ malperfusion, which is obviously due to the nature of the disease^[3,4]^. In aortic dissection surgery, which is actually a palliative treatment method, the aim is to provide perfusion by blocking the false lumen flow and overcoming the malperfusion. Probably the most important problem in ATAAD surgery is finding access for arterial cannulation to maintain inflow line for cardiopulmonary bypass (CBP). Of course, arterial cannulation cannot be performed with conventional methods in a dissected ascending aorta, so peripheral arteries are used for this purpose. Because of the ease of exploration, the most commonly used artery for peripheral cannulation is the femoral artery. If there is a dissection in femoral artery or the possibility of using antegrade cerebral perfusion (ACP) for brain protection, the right axillary artery cannulation is most preferred. In daily practice, we are often come across advantages and disadvantages of these two cannulation methods. For this reason, the results of femoral and axillary cannulation methods used in ATAAD surgery were compared in this study.

## METHODS

Fifty-two patients who underwent emergency surgery for ATAAD between January 2012 and April 2018 were retrospectively evaluated. The patients were grouped as those with femoral arterial cannulation and with axillary arterial cannulation, group 1 (n=22) and group 2 (n=30), respectively. The groups were compared in terms of preoperative, operative and postoperative data.

In our clinic, surgeons’ preference usually comes first in deciding the cannulation method. However, if it is predicted to intervene in the aortic arch and branches by preoperative findings, axillary cannulation is preferred because antegrade cerebral perfusion can be applied with fewer additional cannulae or lines. Another factor affecting our surgeons' preferences when deciding on the cannulation strategy is whether the patient is hemodynamically stable or unstable before the operation. We prefer to cannulate a graft that is anastomosed to the axillary artery in an end-to-side manner instead of direct cannulation, so it takes longer than femoral arterial cannulation. If the patient is not hemodynamically stable, femoral arterial cannulation is preferred as it is easier to explore the femoral artery and perform cannulation. Finally, the presence of dissection in the femoral artery naturally affects our cannulation strategy.

Patients' perioperative and postoperative data were obtained from the hospital registry system and evaluated retrospectively.

Severity was defined by onset of symptoms within 14 days of surgery by taking the medical history. The anatomy of aortic dissection was classified according to the Stanford classification. Patients were retrospectively grouped according to the Penn classification: Penn Class Aa was characterized by absence of ischemic complications, Penn Class Ab by branch vessel malperfusion resulting in localized organ ischemia, Penn Class Ac by circulatory collapse and generalized ischemia and Penn Class Abc by a combination of localized and general ischemia^[5]^. As mentioned in the study of Buonocore et al.^[6]^, we selected for the study only patients in Penn Class Aa to have a population as homogeneous as possible in terms of clinical ischemic profile, with no evidence of preoperative malperfusion, to better discern the possible role of perfusion techniques and cerebral protection strategies on outcomes.

Preoperative variables are: age, gender, comorbidities, previous cardiac surgery, ascending aorta diameter, body surface area, EuroSCORE, hemoglobin level, white blood cell count and ejection fraction. Operative variables are operating time, CPB time, cross-clamp (CC) time, existence of aortic root/arch intervention. Postoperative variables are mortality, new-onset cerebral event, ventilator weaning time, intensive care unit (ICU) stay, hospital stay, postoperative renal failure, amount of blood transfusion and reoperation for bleeding.

All patients underwent transthoracic echocardiography and computed tomography (CT) angiography prior to operation. All patients diagnosed with ATAAD underwent emergency surgery.

A new cerebral event or cerebral infarction on CT scan was identical in both groups.

Postoperative mechanical ventilator dependence over 24 hours was considered as prolonged intubation.

Patients with subacute or chronic type A aortic dissection who underwent elective surgery were not included to our study.

### Surgical Technique in Axillary Arterial Cannulation Group

Monitorization for cerebral and pulse oximetry was used in all patients before induction. Arterial inflow cannula was placed in an 8-mm Dacron graft that is anastomosed to the axillary artery in an end-to-side manner. Following median sternotomy, right atrial venous cannulation and right superior pulmonary venous vent catheter placement were performed. Aortic arch branches were encircled with nylon tapes separately. Aortotomy was performed after CC placement and after aortotomy, the orifices are seen directly in the aortic root and cardioplegia is administered as a single dose by antegrade route. Continuous cardioplegia is administered by retrograde route by the cannula inserted into the coronary sinus. We used isothermic hyperpotassemic blood cardioplegia for all cases. After CC, the patient was cooled to 24-26ºC. The need for aortic root replacement was considered. In other patients, layer between true and false lumen was sandwiched using Bioglue to prepare for proximal anastomosis. Aortic valve was resuspended with 2-0 pledgeted polyester sutures on the commissures. The proximal landing zone was prepared with the sandwich technique. After CC removal, aortic arch pathologies were evaluated during ACP under total circulatory arrest. All branches of the aortic arch were clamped during ACP. Pump flow was fixed to obtain a right radial arterial line pressure of 50-60 mmHg. Aortic arch or distal ascending aorta was prepared for distal anastomosis by the sandwich technique. The distal part of the graft was anastomosed to this distal landing area. The aortic graft was clamped, and the warming period started. The proximal part of the graft was then anastomosed to the previously prepared proximal landing area, or to the distal part of Bentall graft, if any. CPB was discontinued under suitable conditions.

### Surgical Technique in Femoral Arterial Cannulation Group

In this technique, femoral artery was cannulated using the Seldinger technique following femoral exploration. Unlike the axillary arterial cannulation, ACP was performed by using 16F and 14F balloon occlusion catheters for innominate artery and left carotid artery, respectively.

### Endpoints

Primary endpoints compared between the femoral cannulation and axillary cannulation groups were defined following the European Journal of Cardiothoracic Surgery (EJCTS) and Interactive Cardiovascular and Thoracic Surgery (ICVTS) Statistical and Data Reporting Guidelines^[7]^: operative mortality, defined as all-cause mortality at 30 days; occurrence of postoperative neurological injury at 30 days; major adverse cardiac and cerebrovascular events (MACCEs), a composite endpoint of all-cause mortality, myocardial infarction, need for emergency cardiac surgery or percutaneous intervention and stroke up to 30 days postoperatively.

### Statistical Method

Mean, standard deviation, median, lowest, highest, frequency and ratio values were used in the descriptive statistics of the data. The distribution of the variables was measured by the Kolmogorov-Smirnov test. Independent samples *t*-test and Mann-Whitney U test were used in the analysis of independent quantitative data. Chi-square test was used to analyze independent qualitative data, and Fisher’s test was used when the chi-square test conditions were not met. The statistical analysis was performed utilizing SPSS 22.0 software.

## RESULTS

When the preoperative data were evaluated, no statistically significant difference was found between the groups in terms of EuroSCORE or comorbid factors (*P*>0.05) (Table 1).

**Table 1:** Preoperative data.

		Femoral cannulation (group 1) n=22			Axillary cannulation (group 2) n=30			*P*
		Mean±sd/n-%		Med	Mean±sd/n-%		Med	
Age		58.3±13.7		62.0	52.5±15.1		55	0.163^t^
Gender	Female	9	40.9%		16	53.3%		0.376^X2^
	Male	13	59.1%		14	46.7%		
DM		2	9.1%		9	30%		0.068^X^2^^
COPD		6	27.3%		7	23.3%		0.746^X^2^^
Smoking		14	63.6%		16	53.3%		0.458^X^2^^
CRF		2	9.1%		0	0.0%		0.181^X^2^^
HT		15	68.2%		23	76.7%		0.496^X^2^^
Aortic diameter (mm)		54.1±12.2		52.5		56.7±13.5	53	0.690^m^
Marfan		3	13.6%		4	13.3%		0.975^X^2^^
BSA		1.8±0.2		1.8	1.8±0.1		1.8	0.554^t^
Repeat surgery		7	31.8%		5	16.7%		0.200^X^2^^
EuroSCORE		4.6±1.4		4.0	4.6±1.5		5	0.828^m^
CPR		2	9.1%		0	0.0%		0.174^X^2^^
Tamponade or effusion		8	36.4%		8	26.7%		0.454^X^2^^
Hemoglobin (g/dL)		11.4±1.9		11.8	11.6±1.7		11.4	0.690^t^
WBC >11.000 (K/uL)		15	68.2%		15	50%		0.190^X^2^^
EF <50		8	36.4%		9	30%		0.629^X^2^^

^t^: t test; ^m^: Mann-Whitney U test; ^X^2^^: Chi-square test (Fisher's test). BSA=body surface area; COPD=chronic obstructive pulmonary disease; CRF=chronic renal failure; CPR=cardiopulmonary resuscitation; DM=diabetes mellitus; EF=ejection fraction; HT=hypertension; WBC=white blood cells

When the operative variables were analyzed, CPB time, CC time, operating time, ACP time and total circulatory arrest time were the variables that show no statistically significant difference (*P*>0.05). As a result of technique, ACP was performed 100% of patients in group 2, while it was performed in 59.1% in group 1 (*P*<0.05) (because we performed axillary cannulation in all patients for whom we decided to use ACP before the operation) (Table 2).

**Table 2:** Perioperative data.

	Femoral cannulation (group 1) n=22			Axillary cannulation (group 2) n=30			*P*
	Mean±sd/n-%		Med	Mean±sd/n-%		Med	
CPB time (min)	195.0±28.2		199	196.5±31.4		193	0.476^m^
Cross-clamp time (min)	133.3±23.3		134.5	132.4±19.6		133.5	0.897^m^
Operating time (min)	318.2±35		320.5	332.0±74.8		325	0.610^m^
Root intervention	7	31.8%		9	30%		0.888^X^2^^
Hemiarch intervention	7	31.8%		5	16.7%		0.200^X^2^^
Total arch intervention	1	4.5%		5	16.7%		0.176^X^2^^
Only ascending aortic intervention	7	31.8%		12	40%		0.545^X^2^^
ACP	13	59.1%		30	100.0%		*0.000^X^2^^*
ACP time (min)	37.8±16.5		35	40.4±14.9		38.5	0.347^m^
TCA	13	59.1%		22	73.3%		0.279^X^2^^
TCA time (min)	39.5±5.4		39	38.8±4.7		40	0.864^m^
Weaning time (h)	23.3±8.4		25	21.6±15.4		18	0.169^m^
ICU time (days)	7.5±7.1		5	6.4±4.6		5	0.804^m^
Hospital discharge time (days)	13.6±10.7		9	11.7±5.6		10	0.678^m^
Postoperative drainage (ml)	835.7±388.3		800	813.3±380.1		750	0.759^m^
Blood product (IU)	4.7±2.8		4	4.8±2.8		5	0.866^m^
POAKI	8	36.4%		6	20%		0.189^X^2^^
Reoperation for bleeding	7	31.8%		9	30%		0.888^X^2^^
Prolonged intubation	10	45.5%		12	40%		0.584^X^2^^
Neurological complication	5	22.7%		6	20%		0.812^X^2^^
Mortality	3	13.6%		3	10%		0.685^X^2^^

^m^: Mann-Whitney U test; X^2^: Chi-square test (Fisher's test). ACP=antegrade cerebral perfusion; CPB=cardiopulmonary bypass; ICU=intensive care unit; POAKI=postoperative acute kidney injury; TCA=total circulatory arrest

Undoubtedly, the most important parameters that could be potentially affected by cannulation strategies are postoperative mortality and rates of occurrence of new cerebral events. Regarding postoperative variables, there was no statistically significant difference in new cerebral events and mortality. Mortality was 13,6% (n=3) in femoral cannulation group, while it was 10% (n=3) in axillary cannulation group (*P*=0.685). New cerebral event was seen in 5 patients (22.7%) in group 1, while it was seen in 6 patients (20%) in group 2 (*P*=0.812). There was no statistically significant difference in time of weaning from mechanical ventilation, ICU stay, amount of blood transfusion, hospital stay, reoperation for excessive bleeding or acute postoperative kidney injury requiring dialysis (Table 2).

## DISCUSSION

In this single-center retrospective study, emergency operations, performed in ATAAD patients without malperfusion findings before operation, were evaluated. The patients were operated by different surgeons with different surgical strategies for perfusion and cerebral protection. Our aim was to investigate the possible effects of surgical options, especially arterial cannulation preferences, on 30-day mortality and neurological outcomes.

ATAAD patients present very different clinical findings, because ATAAD is an extensive vascular network disease. Due to different presentations, there is a risk that the outcome of clinical trials on ATAAD may be affected. In our study, similar to the study by Buonocore et al.^[6]^, we used the Penn classification and selected only Penn Aa ATAAD patients to remove this risk. As we have already stated in our study, the choice of which method of cannulation should be preferred is based on the surgeon's preferences. However, there are also factors that affect these preferences. As we also mentioned in the methodology section, these factors are in a wide spectrum ranging, like whether ACP is performed or not or the hemodynamic state of the patients. However, in our study, and in other studies, it is seen that these preferences do not affect the homogeneity among the study groups, as the cannulation strategies are compared. For example, Klotz et al.^[8]^, similar to our study, examined the effects of cannulation strategies on postoperative outcomes. In their study, there was a significant difference between groups with peripheral or central cannulation only in terms of ASA score. They reported that the peripheral cannulation group includes patients with higher ASA score. In another study, Buonocore et al.^[6]^ similarly examined the effects of cannulation strategies on outcomes. In this study, the demographic data of the patients were generally similar, only the DeBakey type 2 dissection rate was higher in femoral cannulation group. Although these results in both studies are acceptable, there was no statistically significant difference regarding preoperative data between the groups in our study. This shows that two homogeneous groups were compared, which is the preferred condition for a scientific study.

It arouses curiosity whether cannulation strategies differ in terms of perioperative parameters in ATAAD patients. Generally, it has been shown in many studies that cannulation preferences do not significantly affect operative data. In the study of Klotz et al.^[8]^, they reported that central cannulation was more frequently preferred only in patients undergoing elephant trunk procedure as an additional procedure, and there was no difference between the groups in terms of operative data. The only differences stated by Buonocore et al.^[6]^ are longer total circulatory arrest (TCA) and CC time in femoral arterial cannulation group. In a further study, Etz et al.^[9]^ reported that they detected shorter TCA and operative time in the femoral cannulation group in their study of cannulation strategies in patients with ATAAD. In our study, it may be expected to have longer operation time as we prefer to use a graft anastomosed by end-to-side suturing to axillary artery instead of direct cannulation. However, in this technique, there is no need to repair the axillary artery because of graft ligation just above the anastomosis after decannulation is quite simple. Since the femoral artery is directly cannulated, a significant time is spent for arterial repair after decannulation. For this reason, we think that there is no difference in terms of operating time between the two groups. As we prefer axillary cannulation in all planned ACP patients, the rate of ACP use is naturally lower in the femoral cannulation group. However, we would especially like to emphasize here that, in our patients with femoral cannulation, ACP is provided by suitable strategies in all patients if the branches of the aortic are operated (with an open distal anastomosis).

Undoubtedly, the effect of cannulation strategies on postoperative outcomes in ATAAD surgery is the most curious subject. The study of Buonocore et al.^[6]^, which was basically structured similarly to our study, showed no difference between the groups in terms of postoperative outcomes, mainly postoperative early mortality and postoperative neurological events. Similarly, in Klotz et al.^[8]^ study, cannulation strategies were reported to have no effect in terms of postoperative outcomes. However, many other studies have suggested that axillary cannulation is better than femoral cannulation in terms of early postoperative mortality and long-term outcomes^[9-11]^. In terms of postoperative neurological complications, the discussion is moving toward a more specific point. Some has begun to examine whether ACP is performed unilaterally or bilaterally rather than cannulation strategies^[12,13]^. As it can be seen, the debate on this subject has not finished yet. There are also two meta-analyses on this subject. In one of these meta-analyzes, Ren et al. examined 9 trials involving 715 patients and consequently reported better results on early mortality and neurological complications in the axillary arterial cannulation group^[14]^. Nevertheless, they emphasized that the cannulation strategy should be chosen according to the characteristics of patients in the same meta-analysis. In another meta-analysis, Benedetto et al. examined 8 studies and 793 patients' results^[15]^. And similar to other meta-analyses, they reported that axillary cannulation is superior to femoral cannulation. However, it should be emphasized that these meta-analyses have not been observed enough about whether ACP was used or not, especially in patients with arch intervention. We think this is an important point, because we believe that there would be no difference in terms of postoperative mortality or neurological complications between groups if ACP were performed for cerebral protection. In addition, many studies have shown that the basic parameters affecting mortality and neurological complication rates in ATAAD surgeries are preoperative conditions such as whether the patient has preoperative malperfusion findings^[16-18]^. We think that, in our study, we did not find any difference between the groups in terms of early postoperative data because patients without preoperative malperfusion findings and included in the Penn Aa classification were selected and ACP was performed to every patient in need.

### Limitations of the Study

It is not possible to perform a randomized controlled study on the surgical treatment of ATAAD because is a condition that requires immediate surgical intervention. Our study is not a randomized controlled trial for this reason. On the other hand, the number of cases in our study is small. The reason is that only patients in the Penn Aa classification are included, in particular, to ensure preoperative patient homogeneity. Our results also showed that the preferred cannulation strategies have no effect on results. As mentioned in other studies, we think that the basic parameters affecting the results of ATAAD surgery are mainly preoperative patient characteristics.

## CONCLUSION

In conclusion, we think that the basic parameters affecting the outcomes of ATAAD surgery are the preoperative characteristics of the patients rather than the surgical technique performed. It is also important to note that the use of cerebral protection methods in the required patients is more important than the preferred method of cannulation. For this reason, we believe that patient standardization in the preoperative period with a method like the Penn Aa classification, as we used while assessing the surgical results of ATAAD, will make the study results more reliable.

**Table:** 

Author's roles & responsibilities	
OG	Substantial contributions to the conception or design of the work; or the acquisition, analysis, or interpretation of data for the work; drafting the work or revising it critically for important intellectual content; final approval of the version to be published
LY	Substantial contributions to the conception or design of the work; or the acquisition, analysis, or interpretation of data for the work; drafting the work or revising it critically for important intellectual content; final approval of the version to be published
HI	Acquisition, analysis, or interpretation of data for the work; final approval of the version to be published
NKY	Acquisition, analysis, or interpretation of data for the work; final approval of the version to be published
YB	Acquisition, analysis, or interpretation of data for the work; final approval of the version to be published
SI	Acquisition, analysis, or interpretation of data for the work; final approval of the version to be published
BE	Acquisition, analysis, or interpretation of data for the work; final approval of the version to be published
AG	Acquisition, analysis, or interpretation of data for the work; drafting the work or revising it critically for important intellectual content; final approval of the version to be published

## References

[1] Hiratzka LF, Bakris GL, Beckman JA, Bersin RM, Carr VF, Casey DE Jr (2010). 2010 ACCF/AHA/AATS/ACR/ASA/SCA/SCAI/SIR/STS/SVM Guidelines for the diagnosis and management of patients with thoracic aortic disease: A report of the American college of cardiology foundation/American heart association task force on practice guidelines, American association for thoracic surgery, American college of radiology, American stroke association, Society of cardiovascular anesthesiologists, Society for cardiovascular angiography and interventions, society of interventional radiology, society of thoracic surgeons, and society for vascular medicine. J Am Coll Cardiol.

[2] Mody PS, Wang Y, Geirsson A, Kim N, Desai MM, Gupta A (2014). Trends in aortic dissection hospitalizations, interventions, and outcomes among medicare beneficiaries in the United States, 2000-2011. Circ Cardiovasc Qual Outcomes.

[3] Ertugay S, Bozkaya H, Cinar C, Parildar M, Posacioglu H (2015). Does endovascular repair affect aortic remodeling in acute complicated type B aortic dissection. Turk Gogus Kalp Dama.

[4] Geirsson A, Szeto WY, Pochettino A, McGarvey ML, Keane MG, Woo YJ (2007). Significance of malperfusion syndromes prior to contemporary surgical repair for acute type A dissection: outcomes and need for additional revascularizations. Eur J Cardiothorac Surg.

[5] Augoustides JG, Geirsson A, Szeto WY, Walsh EK, Cornelius B, Pochettino A (2009). Observational study of mortality risk stratification by ischemic presentation in patients with acute type A aortic dissection. Nat Clin Pract Cardiovasc Med.

[6] Buonocore M, Amarelli C, Scardone M, Caiazzo A, Petrone G, Majello L (2016). Cerebral perfusion issues in acute type A aortic dissection without preoperative malperfusion: how do surgical factors affect outcomes?. Eur J Cardiothorac Surg.

[7] Hickey GL, Dunning J, Seifert B, Sodeck G, Carr MJ, Burger HU (2015). Statistical and data reporting guidelines for the European journal of cardiothoracic surgery and the interactive cardiovascular and thoracic surgery. Eur J Cardiothorac Surg.

[8] Klotz S, Heuermann K, Hanke T, Petersen M, Sievers HH (2015). Outcome with peripheral versus central cannulation in acute Type A dissection. Interact Cardiovasc Thorac Surg.

[9] Etz CD, von Aspern K, da Rocha E Silva J, Girrbach FF, Leontyev S, Luehr M (2014). Impact of perfusion strategy on outcome after repair for acute type a aortic dissection. Ann Thorac Surg.

[10] Svensson LG, Blackstone EH, Rajeswaran J, Sabik JF III, Lytle BW, Gonzalez-Stawinski G (2004). Does the arterial cannulation site for circulatory arrest influence stroke risk. Ann Thorac Surg.

[11] Tiwari KK, Murzi M, Bevilacqua S, Glauber M (2010). Which cannulation (ascending aortic cannulation or peripheral arterial cannulation) is better for acute type A aortic dissection surgery. Interact CardioVasc Thorac Surg.

[12] Zierer A, Risteski P, El-Sayed Ahmad A, Moritz A, Diegeler A, Urbanski PP (2014). The impact of unilateral versus bilateral antegrade cerebral perfusion on surgical outcomes after aortic arch replacement: a propensity-matched analysis. J Thorac Cardiovasc Surg.

[13] De Paulis R, Czerny M, Weltert L, Bavaria J, Borger MA, Carrel TP (2015). Current trends in cannulation and neuroprotection during surgery of the aortic arch in Europe. Eur J Cardiothorac Surg.

[14] Ren Z, Wang Z, Hu R, Wu H, Deng H, Zhou Z (2015). Which cannulation (axillary cannulation or femoral cannulation) is better for acute type A aortic dissection repair? A meta-analysis of nine clinical studies. Eur J Cardiothorac Surg.

[15] Benedetto U, Mohamed H, Vitulli P, Petrou M (2015). Axillary versus femoral arterial cannulation in type A acute aortic dissection: evidence from a meta analysis of comparative studies and adjusted risk estimates. Eur J Cardiothorac Surg.

[16] Trimarchi S, Nienaber CA, Rampoldi V, Myrmel T, Suzuki T, Mehta RH (2005). Contemporary results of surgery in acute type A aortic dissection: the international registry of acute aortic dissection experience. J Thorac Cardiovasc Surg.

[17] Hagan PG, Nienaber CA, Isselbacher EM, Bruckman D, Karavite DJ, Russman PL (2000). The international registry for aortic dissection (IRAD). New insights into an old disease. JAMA.

[18] Grimm JC, Magruder JT, Crawford TC, Sciortino CM, Zehr KJ, Mandal K (2016). Differential outcomes of type A dissection with malperfusion according to affected organ system. Ann Cardiothorac Surg.

